# Statins, fibrates, thiazolidinediones and resveratrol as adjunctive therapies in sepsis: could mitochondria be a common target?

**DOI:** 10.1186/2197-425X-2-9

**Published:** 2014-04-17

**Authors:** Jerome Morel, Mervyn Singer

**Affiliations:** Département d’anesthésie réanimation, Centre Hospitalier Universitaire de Saint Etienne, 42055 Saint Etienne, France; Bloomsbury Institute of Intensive Care Medicine, University College London, London, WC1E 6BT UK

**Keywords:** Statin, Fibrates, Thiazolidinedione, Resveratrol, Mitochondria, Sepsis, Pleiotropic effects, Peroxisome proliferator-activated receptors

## Abstract

Through their pleiotropic actions, statins, fibrates, thiazolidinediones and resveratrol can target multiple mechanisms involved in sepsis. Their actions on mitochondrial function are of interest in a pathological state where bioenergetic failure may play a key role in the development of organ dysfunction. We review these four drug groups as potential adjunctive therapies in sepsis with a particular focus upon mitochondria. Systematic review of clinical and experimental trials was done with a literature search using the PubMed database. Search terms included statins, fibrates, thiazolidinediones, resveratrol, mitochondria, sepsis, peroxisome proliferator-activated receptors, inflammation, oxidative stress and organ dysfunction. With the exception of statins, most of the compelling evidence for the use of these agents in sepsis comes from the experimental literature. The agents all exert anti-inflammatory and anti-oxidant properties, plus protective effects against mitochondrial dysfunction and stimulation of mitochondrial biogenesis. Improved outcomes (organ dysfunction, survival) have been reported in a variety of sepsis models. Notably, positive outcome effects were more commonly seen when the agents were given as pre- rather than post-treatment of sepsis. Statins, fibrates, thiazolidinediones and resveratrol prevent sepsis-induced injury to organs and organelles with outcome improvements. Their effects on mitochondrial function may be integral in offering this protection. Definitive clinical trials are needed to evaluate their utility in septic patients or those at high risk of developing sepsis.

## Review

### Introduction

Severe sepsis is characterized by a dysregulated systemic inflammatory response to infection resulting in acute multiple organ dysfunction (MOF) and a high mortality rate. The pathophysiology of sepsis-induced MOF remains incompletely understood but a growing body of evidence supports impairment of cellular oxygen utilization as a key mechanism [1–4]. Considerable enthusiasm has recently surrounded the potential beneficial effect of fibrates, thiazolidinediones (TZD) and, particularly, statins as adjunctive therapies for sepsis [5–9]. Some experimental studies also suggest a role for resveratrol [10–14]. However, most of these positive outcomes have been generated in laboratory models of sepsis such as caecal ligature and puncture or injection of endotoxin. The discrepancies observed to date between human and experimental studies may relate to the difficulty in reproducing the complexity of human sepsis and/or the use of doses far in excess of those currently used in clinical practice*.* Prospective clinical trial data are insufficient, particularly for fibrates and TZDs [15–17], and non-existent for resveratrol.

Statins, fibrates and TZDs modulate lipid and glucose metabolism. Resveratrol, a phenol constituent of red wine, is not available as a stand-alone medication but has been reported to slow down carcinogenesis, cardiovascular disease and ischaemic injury [18]. All four classes exert pleiotropic effects through mechanisms that remain incompletely understood [6, 8, 18, 19]. Immune-inflammatory modulation is a common property; however, several authors have also underlined effects on mitochondrial function [14, 20–23]. This may be highly pertinent to critical illness as bioenergetic dysfunction is implicated in the pathophysiology of sepsis-induced multi-organ failure [4].

In view of this burgeoning interest, it is timely to summarize the main recognized mechanisms of these agents, including their actions on mitochondria, and to offer a critical review of currently available experimental and clinical data.

#### Modes of action

##### Statins

**Figure 1:**
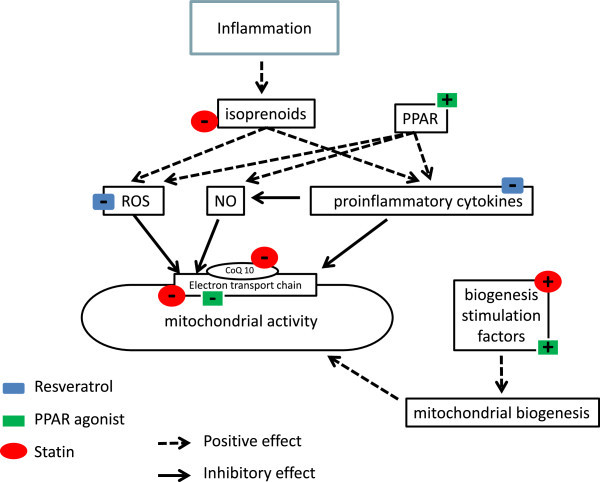
**Schema showing mechanisms involved in mitochondrial dysfunction.** During sepsis and potential points of modulation by statins, PPAR agonists and resveratrol. ROS, reactive oxygen species; NO, nitric oxide; PPAR, peroxisome proliferator-activated receptor; CoQ10, coenzyme Q10, ubiquinone.

In addition to lowering low-density lipoprotein (LDL) cholesterol, statins exhibit a wide range of other biological effects. Statins inhibit 3-hydroxy-3-methyl-glutaryl-coenzymeA (HMGCoA) reductase, a key enzyme in the mevalonate pathway. Mevalonate is a precursor for cholesterol, ubiquinone and isoprenoids (Figure 1) [24]. Thus, statins can decrease all three end products but, as a consequence of enzyme affinity, mainly reduce cholesterol production.

Ubiquinone is both an electron carrier within the mitochondrial electron transport chain that generates ATP and a powerful anti-oxidant [21]. While several clinical and experimental studies have reported that statins decreased the ratio of plasma ubiquinone to LDL (its natural carrier), its effects on tissue ubiquinone levels are more controversial. It may be an important mechanism underlying statin-induced myopathy [25].

Isoprenoids (farnesyl pyrophosphate and geranyl pyrophosphate) serve as lipid attachments and activators for various signalling molecules such as G-protein and GTP-binding protein (Ras and Ras-like protein) [24], which have been associated with reactive oxygen species (ROS) production and activation of pro-inflammatory pathways (reviewed by Blanco-Colio et al. [26]). The pleiotropic effects of statins have been associated with decreased levels of these small proteins [27]. Both *in vivo* and *in vitro* studies show that statins can induce cellular accumulation of endothelial nitric oxide synthase, inhibit expression of adhesion molecules and chemokines that recruit inflammatory cells, inhibit expression of pro-coagulant factors, induce production of anti-coagulant substances, increase apoptosis, decrease oxidative stress, and modulate the adaptive immune system (reviewed by Terblanche et al. [8]). In a volunteer study, pre-treatment with simvastatin prior to lipopolysaccharide (LPS) attenuated the upregulation of Toll-like receptor 2 and 4 surface expression on circulating monocytes [28]. How many of these effects are related to lowering LDL cholesterol remains uncertain. Of note, squalestatin, a selective inhibitor of the synthesis of sterol derivatives, has no anti-inflammatory effect compared to statins [24].

Statins can affect skeletal muscle mitochondria *in vitro* by inhibiting respiratory chain complexes and oxidative capacity [29, 30], decreasing mitochondrial membrane potential [30], uncoupling oxidative phosphorylation [30], inducing mitochondrial swelling and apoptosis [30] and decreasing mitochondrial density [31] (Figure 1). However, no clear relationship has been documented between a decrease in ubiquinone and alterations in mitochondrial function. Hydrophilic statins (e.g. pravastatin) are much less ‘mitotoxic’ compared to lipophilic statins such as cerivastatin, fluvastatin, atorvastatin and simvastatin [30]. It is noteworthy that the toxic effects of atorvastatin are mostly observed with doses that are much higher than those prescribed to patients. The delayed metabolism of statins seen in critical illness may result in very high plasma levels so the risk of toxicity would potentially increase; however, this would be difficult to distinguish in an unstable patient with concurrent multi-organ failure [32]. As discussed later, these ‘toxic’ effects may, paradoxically, offer some protective effects during sepsis. Recently, Bouitbir et al. reported that statin-treated patients who underwent cardiac surgery had decreased oxidative stress, enhanced oxidative capacity, and a marked augmentation of mRNA expression of the peroxisome proliferator-activated receptor (PPAR) gamma co-activators 1α *(PGC-1α)* and 1β *(PGC-1β)*. PGC-1α is the main regulator of mitochondrial biogenesis, i.e. new protein turnover [20]. This study raises new insights regarding the action of statins, but the clinical impact remains to be explored.

##### Fibrates and thiazolidinediones

PPARs are ligand-activated transcription factors that belong to the nuclear receptor superfamily. Once activated by ligands, PPARs form a heterodimer with the retinoic X receptor (RXR) that allows recruitment of a set of co-activators or co-repressors [6, 33]. This heterodimer binds to the PPAR response element within the promoter region of its target genes, provoking either expression or repression. PPAR inhibits expression of pro-inflammatory cytokines through direct or indirect actions on pro-inflammatory transcription factors (NF-κB, STAT, AP-1) [6, 34].

Fibrates are synthetic ligands of peroxisome proliferator-activated receptor-α (PPAR-α). Fibrates are used for treating dyslipidaemias, lowering both triglyceride and LDL cholesterol levels. They also ameliorate insulin resistance and glucose intolerance [5, 35]. The PPAR-α receptor is expressed mainly in brown fat and liver but has been found in many other cells [5, 36].

TZDs are synthetic ligands of peroxisome proliferator-activated receptor-γ (PPAR-γ). While most of their effects appear dependent upon PPAR activation, TZDs could also exert anti-inflammatory effects in macrophages via a PPAR-independent pathway [37]. Thiazolidinediones are drugs used for managing type II diabetes mellitus and the metabolic syndrome. Rosiglitazone, pioglitazone, troglitazone, rivoglitazone and ciglitazone are members of this therapeutic class. However, most have been withdrawn from the market due to adverse effects on heart. The PPAR-γ receptor is highly expressed in adipose tissue and, to a lesser extent, in intestine, immune and stem cells [6]. Activation of these receptors decreases insulin resistance and modifies lipid storage. A time-dependent downregulation of PPAR-γ expression has been reported in experimental sepsis that is partially restored by TZDs [34].

After various inflammatory insults, *in vivo* and *in vitro* studies have shown that both fibrates and TZDs improved endothelial dysfunction [38, 39], inhibited expression of adhesion molecules and inflammatory cytokines [40, 41] and decreased oxidative stress and nitric oxide production [39, 42]. Fibrates can inhibit coagulation [38, 43] and may improve haemorheologic parameters [44]. TZDs increase plasma adiponectin levels [45] and may initiate macrophage apoptosis via caspase-3 activation [46].

Both drug groups also impair mitochondrial function, at least *in vitro*, via direct inhibition of mitochondrial respiration (mainly complex I) [47], by membrane depolarization [48] and through increases in uncoupled respiration [49, 50] (Figure 1). At lower doses, TZDs enhanced mitochondrial potential and promoted lymphocyte survival [51]. Several studies report that PPAR-γ agonists induce mitochondrial biogenesis by mechanisms that are not fully understood but could be mediated, at least in part, via activation of PGC-1α [52, 53]. None of these studies have been carried out in sepsis, where early activation of mitochondrial biogenesis has been associated with survival [54].

Statins, fibrates and TZDs can act synergistically to affect some of the pathways previously described [55]. For example, patients with cardiovascular disease showed additive anti-inflammatory effect when statins were given in combination with PPAR-γ agonists [56].

##### Resveratrol

Resveratrol is a natural phenol present in many plants but especially abundant in red grapes, peanuts and mulberries [18]. It exerts beneficial effects in experimental sepsis when administered either before or shortly after the septic insult. The mechanisms involved are not yet clearly defined. Recent research has demonstrated that resveratrol activates the silent mating type information regulator 2 homolog 1 (SIRT1), which is a key regulator of cellular defenses and cell survival in response to stress [57]. Of specific interest in sepsis is the interaction between SIRT1 and mitochondrial biogenesis [23, 57]. Resveratrol has also been shown to downregulate the pro-inflammatory response [14, 58] and to have anti-oxidant properties [11, 57].

#### Experimental and clinical evidence of statins, fibrates and thiazolidinedione effects in sepsis

##### Statins

In a murine model of sepsis, simvastatin pre-treatment markedly improved survival times (median 128 h versus 28 h, *p* < 0.005) [59]. Even treatment commencing after the onset of sepsis improved survival times, though less impressively (median 37 h versus 23 h for placebo, *p* < 0.05) [60]. In another study, 3 days of simvastatin pre-treatment improved survival and reduced sepsis-induced acute kidney injury by direct effects on the renal microvasculature, reversal of tubular hypoxia and a decrease in systemic inflammation [61]. In a rat model of peritonitis, 30 days' pre-treatment with high doses of simvastatin or atorvastatin prevented hepatic mitochondrial enzyme dysfunction; however, no improvement was seen in liver dysfunction while mortality differences were not reported [62].

Several observational studies have reported significant survival improvement in large cohorts of patients on statin therapy who suffer from bacterial [63–66] or viral infections [67]. However, some authors argue this simply represents a healthy user effect [68, 69]. An association with harm was even reported in a study of infections post-stroke [70]. Four double-blind, placebo-controlled, randomized clinical studies have been performed to examine the impact of either introducing [71–73] or continuing [74] statin therapy in patients with sepsis. Statin treatment was not associated with a significant decrease in pro-inflammatory markers compared to placebo [71, 73, 74]. In a different context, healthy volunteers randomized to statin pre-treatment and subsequently challenged with inhaled LPS manifested less pulmonary and systemic inflammation [75]. While one study showed a reduction in the progression to severe sepsis in patients taking statins, albeit with a similar rate of intensive care admissions between the statin and control groups [72], this was not confirmed by other studies [71, 73, 74]. Finally, stopping statin therapy in septic patients was not associated with worse outcomes [74]. As mentioned previously, statins should be used with caution in critically ill patients due to their unpredictable pharmacokinetics and the amplifying effect related to co-administration of drugs with cytochrome P450 inhibitory effects [32]. Opinions on the utility of statins in sepsis thus remain mixed.

##### Fibrates

A few studies have reported improved outcomes following fibrate therapy in experimental sepsis [38, 76]. For example, influenza-infected mice pre-treated with gemfibrozil had a 54% reduction in mortality [76]. In patients with chronic hepatitis C and hyperlipidaemia, bezafibrate given as adjunctive therapy decreased plasma virus titres and improved liver dysfunction [16]. Fibrates also prevented muscular atrophy [76], a major problem occurring with sepsis. Protective mechanisms need to be elucidated but a decrease in atrogen and myostatin expression has been shown with fibrates therapy in a rat model of chronic inflammation [77].

Children with severe sepsis had decreased leukocyte PPAR-α expression, and this was related to disease severity [78]. In a randomized controlled trial in children with severe burn injury, in which condition mitochondrial dysfunction has been previously observed [79], mitochondrial biogenesis and oxidative phosphorylation were improved with fenofibrates therapy commenced within a week post-burn [35].

##### Thiazolidinediones

In different models of sepsis, pre- or post-treatment with TZD improved outcomes, blunted pro-inflammatory cytokine production and reduced organ injury [34, 42, 45, 80, 81]. A recent study in endotoxic mice revealed protection from the PPAR-γ agonist, rosiglitazone with less reduction in mitochondrial content, improved cardiac dysfunction and better survival rates [82]. On the other hand, the PPAR-α agonist WY-14643 offered no protection. Pioglitazone given to healthy volunteers after endotoxin challenge did not affect plasma levels of TNFα, IL-6 levels or the adhesion molecule VCAM ([83] abstract). An improvement in blood pressure was reported in one study investigating ciglitazone administration following caecal ligation and puncture in rats, but there was no effect on survival [34]. No observational clinical trials are available with TZDs; however, users appear to be at higher risk of pneumonia or lower respiratory tract infection [84]. The risk of heart attack described with this drug has discouraged its widespread use and thus the possibility of conducting large-scale observational studies [85]. Several studies reported a significant reduction in weight loss with TZDs following different types of infectious challenge [42, 86, 87]. This observation has not been specifically studied but TZDs block activation of NF-κB [34], a main activator of muscle wasting [88], in addition to their effects on stimulating PGC-1α and mitochondrial biogenesis [89].

The role of TDZs is not limited to bacterial infection. In a randomized placebo-controlled trial of 140 patients suffering from non-severe malaria, Boggild et al. reported in faster blood parasite clearance and a more rapid decrease in inflammatory biomarker levels (IL-6 and monocyte chemoattractant protein-1) in those given rosiglitazone as adjunctive therapy [15]. After a lethal dose of *C. Albicans*, mice post-treated with pioglitazone had better outcomes and less renal dysfunction [87]. Rosiglitazone also dramatically improved survival in a murine influenza model [86, 90] and reduced HIV-1 replication in lymphocytes and brain macrophages in an experimental model of HIV-1 encephalitis [91].

##### Resveratrol

Resveratrol improved sepsis-induced acute organ injury [10, 12, 13], but the effect on mortality was uncertain [12, 14]. In different cell or tissue types, resveratrol upregulated PGC-1α and decreased mitochondrial ROS production with a consequent increase in mitochondrial size, DNA content and mitochondrial respiratory enzymatic activity [14, 23].

## Conclusions

Through their pleiotropic actions, statins, fibrates, thiazolidinediones and resveratrol can target multiple mechanisms involved in sepsis. These are summarized in the schema shown in Figure 1. Their actions on mitochondrial function and, particularly mitochondrial biogenesis, are of interest in a pathological state where mitochondrial dysfunction may play a key role in the development of organ dysfunction. We can speculate that inhibitory effects of these agents, in particular statins, on mitochondrial function in otherwise healthy patients may potentially offer some benefit in the septic state. By decreasing mitochondrial activity and membrane potential, production of mitochondrial reactive oxygen species would decrease and this may result in a greater degree of cell and mitochondrial protection. Further clinical and experimental studies are warranted to reveal whether benefit can be shown in septic patient populations or in those at high risk of developing sepsis.

## Abbreviations

HMGCoA: 3-hydroxy-3-methyl-glutaryl-coenzymeA
LDL: low-density lipoprotein
LPS: lipopolysaccharide
PGC-1β: peroxisome proliferator activated receptor γ coactivator 1 β
PGC-1α: peroxisome proliferator activated receptor γ coactivator 1 α
PPAR: peroxisome proliferator-activated receptor
TZD: thiazolidinediones
ROS: reactive oxygen species
SIRT1: silent mating type information regulator 2 homolog-1.
